# *KIF14* and *E2F3* mRNA expression in human retinoblastoma and its phenotype association

**Published:** 2009-01-26

**Authors:** Jagadeesan Madhavan, Moutushy Mitra, Kandalam Mallikarjuna, Oberoi Pranav, Ramalingam Srinivasan, Amit Nagpal, Perumal Venkatesan, Govindasamy Kumaramanickavel

**Affiliations:** 1 Sankara Nethralaya Oil and Natural Gas Corporation (SNONGC) Department of Genetics and Molecular Biology, Vision Research Foundation, Sankara Nethralaya, Chennai, India; 2 Larsen and Toubro Ocular Pathology Department, Vision Research Foundation, Sankara Nethralaya, Chennai, India; 3 Department of Biotechnology, National Brain Research Centre, Manesar, India; 4 Tuberculosis Research Centre, Indian Council of Medical Research (ICMR) Chetpet, Chennai, India; 5 Shri Bhagwan Mahavir Vitreo Retina Services, Medical Research Foundation, Sankara Nethralaya, Chennai, India

## Abstract

**Purpose:**

We quantified mRNA expression of candidate genes for proliferation (*KIF14* and *E2F3*) in a large retinoblastoma tumor cohort and associated with disease phenotype.

**Methods:**

*KIF14* and *E2F3* mRNA expression was quantified by real time PCR in 57 retinoblastoma (RB) tumors, 3 RB cell lines, and control samples that included 4 each fetal, age-matched, adult retinas. Immunohistochemistry was done to confirm KIF14 and E2F3 protein expression in tumor cells. The mRNA expression levels were correlated with disease phenotypes including the significance of chemotherapy on tumors.

**Results:**

There was statistically significant overexpression of *KIF14* and *E2F3* mRNA in tumors compared with control retinas (p<0.0001). Further, *E2F3* also showed a significant overexpression compared to RB cell lines (p=0.01). Immunohistochemistry confirmed KIF14 and E2F3 protein overexpression in tumor cells. *KIF14* had significant mRNA overexpression with older age (p=0.01) in presenting patients and in unilateral RB patients (p=0.04). Chemotherapy-treated tumors showed a significant decrease in *KIF14* and *E2F3* expression compared to untreated tumors (p<0.01 and 0.001, respectively).

**Conclusions:**

This report confirms significant mRNA overexpression of *KIF14* and *E2F3* together in a large cohort of RB tumors. The decreased expression in chemotherapy treated cases needs further validation in a large chemotherapy-treated cohort.

## Introduction

Recent advancements in retinoblastoma (RB) research yielded vital information on additional events after two hits in RB progression. 1q31–32 (62%) and 6p22 (43%) are the regions more widely shown to cause gain of site in RB tumors [1]. Studies have shown the *KIF14* gene in the 1q31–32 region [2] and the *E2F3/DEK* genes in the 6p22 region [3,4] are the potential candidate genes that cause the gain of site.

*KIF14*, a mitotic kinesin gene, has been predicted to be a possible oncogene in the 1q region, and was found to overexpress by more than 2 orders of magnitude in RB [2]. Further, *KIF14* overexpression has a strong correlation with age at diagnosis in RB, and probably represents the amount of chromosomal/genetic instability required for tumor formation [5]. The level of KIF14 has been correlated with mitotic progression in the cell cycle, and this protein, along with the microtubule-bundling protein PRC1 and citron kinase, with which it interacts, plays an important role in cytokinesis during midbody formation and completion of cytokinesis [6].

E2F, in conjunction with its dimerization partner, regulates genes that play a role in DNA replication [7,8]. Reports show that tumors with complete gain at loci on chromosome 6p were diagnosed significantly later with a median age at diagnosis [3]. Chromosome 6p gain is also common in bladder cancer and associated with an elevated risk of progression of bladder cancer [9]. The objective of the present work was to quantify the mRNA expression of *KIF14* in a large cohort of 57 RB tumors, which includes 28 tumor samples previously reported by us ( [5]; used with publisher’s permission), along with *E2F3*, in the same cohort, and do a phenotype correlation.

## Methods

### Clinical samples

The study adhered to the Declaration of Helsinki. This study was conducted at the Medical Research Foundation and Vision Research Foundation, Sankara Nethralaya, India, and was approved by the institutional ethics boards. Informed consent was obtained from the parents of RB patients for the research use of RB tumor samples obtained from enucleated eyes removed as a part of treatment. Normal human retinas were obtained from the C.U. Shah Eye Bank (Medical Research Foundation, Sankara Nethralaya, Chennai) and the Lions Eye Bank (The Regional Institute of Ophthalmology, Chennai), after proper approval and the eyeballs were examined under the microscope to rule out any pre-existing pathology. Two fetal retinas were commercially purchased (Advanced Bioscience Resources, Inc., Alameda, CA), after proper ethical clearance and 2 were gifted by Rod Bremner, (Toronto Western Research Institute, Toronto,Canada). In total, 57 RB tumor samples were collected from eyes removed as a part of therapy in patients with age at diagnosis between 1 to 84 months. Out of the 57 samples, 2 samples were obtained from patients who underwent chemotherapy before enucleation. Further, out of 57 samples studied, *KIF14* mRNA expression data of 28 samples previously published by us [5] were selected for this study.

### RNA extraction and reverse transcription

Total RNA was extracted from tumors and normal healthy retina by the guanidine isothiocyanate and chloroform method (TRI Reagent®; Sigma Aldrich, Bangalore, India) as per manufacturer’s instructions. All RNA samples were treated with Turbo DNase® (Ambion, Genetix Biotech Asia Pvt. Ltd, Chennai, India). For all samples, 1 μg total RNA was used to synthesize first-strand cDNA using SuperScript II® reverse transcriptase (Invitrogen, Joyvel, Chennai, India) and random primers.

### Real-time RT–PCR analyses

TaqMan gene expression assays were used to quantify the mRNA expression of *KIF14* (Hs00978216_m1) and *E2F3* (Hs00605457_m1) normalized against two endogenous controls: *GAPDH* (Hs99999905_m1) and *HPR*T (Hs99999909_m1; Applied Biosystems, LabIndia, Chennai, India). Quantification of gene expression was performed in triplicate in a 20 μl volume in 96 well plates on an ABI prism 7300 real time PCR System. Each reaction included 1X TaqMan primer probe mix, 1X TaqMan Universal PCR Master Mix, and 100 ng cDNA. Cycling conditions were as follows: 2 min at 50 °C, 10 min at 95 °C, and 40 cycles of 15 s at 95 °C plus 1 min at 60 °C. The normalized gene-of-interest (GOI) expression levels were calculated as follows: C_t_ values were transformed to quantities by using the comparative C_t_ method. Here, the calibrator’s (F2) quantities for each gene were set to 1. The normalization factor for each sample was calculated by taking the geometric mean of the two housekeeping genes (*GAPDH* and *HPRT*) using geNorm software. The normalized GOI expression levels were calculated by dividing the raw GOI quantities for each sample by the appropriate normalization factor.

### Immunohistochemistry

Paraffin sections of RB tumors and non-neoplastic retina from a 52-year-old donor’s eyeball (5 µm thick) were dewaxed and rehydrated. Antigen retrieval was performed to unmask the antigens for better antigen antibody interactions. Following the dewaxing and rehydration protocol, the slides were immersed with preheated citrate buffer (0.1 M Tri sodium citrate and 0.1 M citric acid with a pH 6.0) in the pressure cooker on the hot plate. The slides were exposed to steam inside pressure cooker until two whistles are completed. Immediately, the pressure cooker was subjected to cooling under running tap water. Endogenous peroxidase activity was blocked for 10 min with 3% H_2_O_2_ in H_2_O, and the slides were incubated with 1:75 rabbit polyclonal affinity purified anti-*KIF14* antibody in Tris buffer, pH 7.6 (BL358; Bethyl Laboratories, Genuine Chemical Corp, India) and with 1:75 mouse monoclonal anti-E2F3 antibody in Tris buffer, pH 7.6 (Upstate, Millipore, Billerica, MA), a kind gift from Rod Bremner (Toronto Western Research Institute, Toronto, Canada) separately. Immunostaining was performed using Dako LSAB+system-horseradish peroxidase (Dakocytomation, Glostrup, Denmark). The reaction was revealed by 3, 3′-diaminobenzidine tetrahydrochloride (Dakocytomation) and counterstained with hematoxylin. For the negative control, immunostaining was done without primary antibody.

### Immunoanalysis

Evaluation of immunostaining in tumor cells was objectively performed by two investigators (J.M. and K.M.) in 6 tumors from the cohort. Ten tumor fields were randomly scanned for protein expression under 40X, and percentage of positive tumor cells was noted for each field. Finally, the average expression was calculated for the entire slide from the 10 values. Depending on the percentage of positive cells, 4 categories were established: 0, no positive cells; 1+, positive cells in less than one-third; 2+, positive cells in 33%–67% and 3+, positive cells in more than two-thirds of total tumor cell population [10].

### Statistical analysis

mRNA expression levels of *KIF14* and *E2F3* in tumors were separately compared to those in RB cell lines, fetal, age-matched and adult retinal controls using the Mann–Whitney U test. Associations between phenotypic characteristics (median age at diagnosis and disease duration) were also studied by Pearson’s coefficient of correlation. The influence of familial tumors, laterality, differentiation, and invasion on gene expression was assessed. The expression levels in tumors exposed to chemotherapy were analyzed separately for significance against untreated tumor samples. All analyses were performed with SPSS version 13.0 (SPSS Inc., Chicago, IL) and p<0.05 was considered significant.

## Results

### Quantification of *KIF14* and *E2F3* mRNA expression levels in tumor and control samples

**Figure 1:**
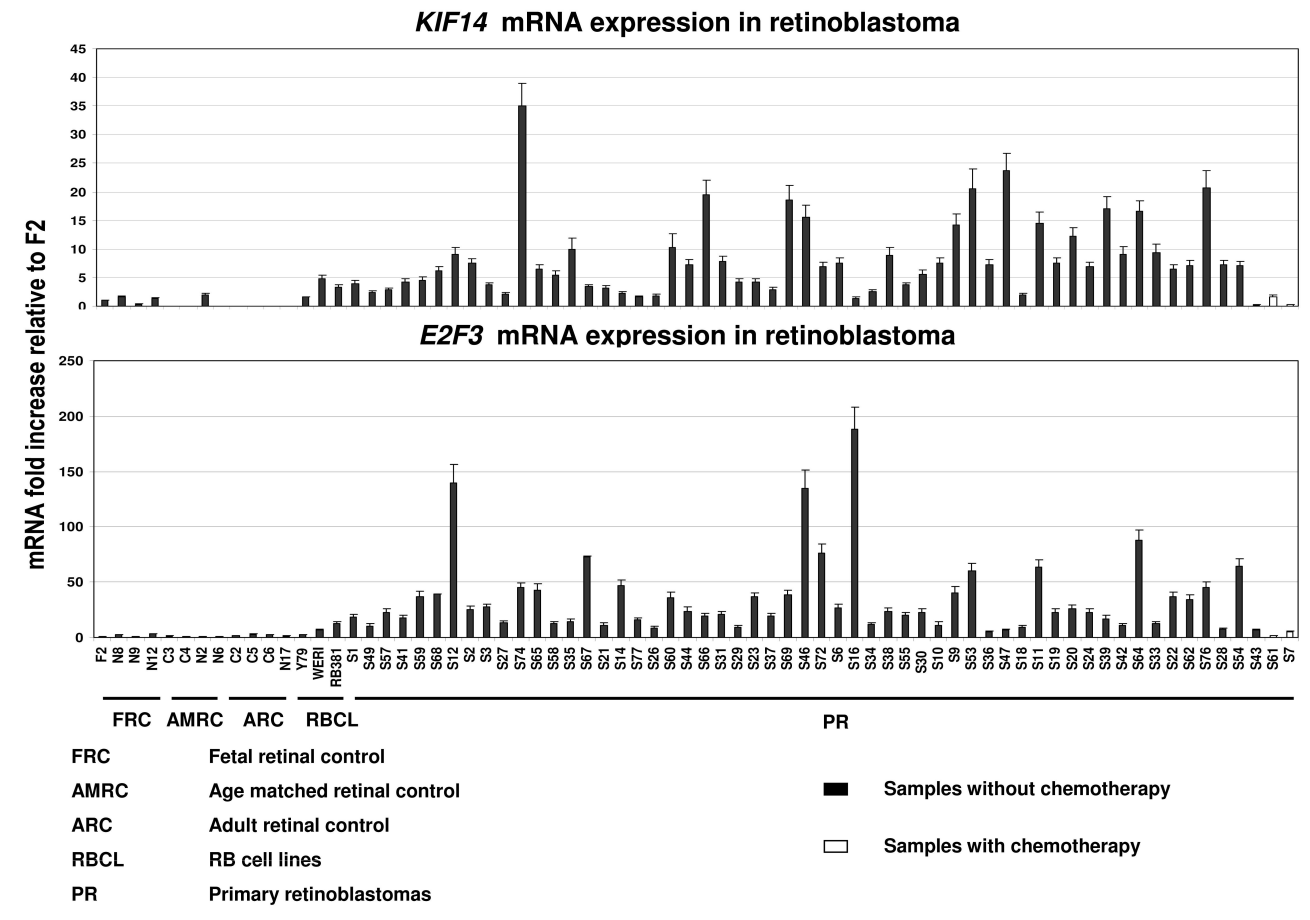
Quantification of *KIF14* and *E2F3* mRNA expression levels in tumor and control samples calibrated to fetal control retina (F2). There was statistically significant overexpression of *KIF14* and *E2F3* mRNA expression in tumors compared to fetal retinal, age-matched and adult control retinas (p<0.0001). There was a significant overexpression of *E2F3* expression compared to RB cell line controls (p=0.01). The chemotherapy-treated subset (last two unshaded bars) showed a significant decrease in the expression of *KIF14* (p<0.01) and *E2F3* (p<0.001) compared to untreated tumors. Error bars represent standard error of the relative fold expression levels of *KIF14* and *E2F3*.

We quantified *KIF14* and *E2F3* mRNA in 57 tumors samples, of which 2 had received chemotherapy before enucleation (Figure 1). For controls, *KIF14* and *E2F3* mRNA levels were determined in 4 fetal (18–20-weeks gestation), 4 age-matched (13–18 months old), 4 adult retinas (21–60 years), and 3 RB cell lines (Y79, WERI, and RB381). Levels were normalized to 2 housekeeping genes, *GAPDH* and *HPRT*. The average fold change between all tumors, as well as the subsets of chemotherapy untreated tumors with each control set was calculated, as well as that between the untreated and treated tumor sets (Table 1). There was statistically significant overexpression of *KIF14* and *E2F3* mRNA expression in tumors compared to fetal retinal, age-matched and adult control retinas (p<0.0001). There was a significant overexpression of *E2F3* expression compared to RB cell line controls (p=0.01). The chemotherapy-treated subset showed a significant decrease in the expression of *KIF14* (p<0.01) and *E2F3* (p<0.001) compared to untreated tumors.

**Table 1:** *KIF14* and *E2F3* mRNA expression in tumors compared to control samples.

**Control**	**Gene expression**	**Average fold change^a^**	**p value**	**Increased**		**Decreased**	
**# of tumors**	**Fold up^a^**	**# of tumors**	**Fold down^a^**
Fetal retina compared to chemotherapy untreated tumors	*KIF14*	7.0	<0.0001*	57	1.1–29.1	0	-
	*E2F3*	20.4		57	3–110.7	0	
Age matched retina compared to chemotherapy untreated tumors	*KIF14*	239.7		57	40–1000	0	-
	*E2F3*	50.3		57	1.1–280.5	0	
Adult retina compared to chemotherapy untreated tumors	*KIF14*^b^	-		-	-	-	-
	*E2F3*	17.8		56	2.6–96.4	1	1.1
Cell lines compared to chemotherapy untreated tumors	*KIF14*	2.5	0.13	44	1.1–10.6	13	1–2.3
	*E2F3*	5.0	0.01*	54	1.1–27.3	3	1–1.3
Tumor after chemotherapy compared to chemotherapy untreated tumors	*KIF14*	34.9	0.01*	53	1.3–23.7	2	1.1–3.5
	*E2F3*	5.8	0.001*	55	5.8–145.8	0	-

Statistical significance of *KIF14* and *E2F3* mRNA expression in tumors compared to control samples. ^a^Fold change is expressed relative to the median of the control samples. ^b^Shows infinity value against adult retina controls. *Statistically significant.

### Phenotype correlation

We examined whether there was a correlation between *KIF14* and *E2F3* mRNA expression levels in the 55 untreated tumors and various tumor phenotypes (Table 2). Samples from the 2 patients who received chemotherapy before enucleation were excluded from further analysis. There was a statistical increase in *KIF14* expression with older age at presentation (p=0.01) and in unilateral RB patients (p=0.04). No correlation was found between *KIF14* mRNA expression and differentiation, familial tumors, invasion of the tumor into the choroid and optic nerve, or the duration of the disease (defined as the period between identification of symptoms to the time of enucleation). Further, there was no correlation between the mRNA expression of *E2F3* and different tumor phenotypes (Table 2).

**Table 2:** Significance of *KIF14* and *E2F3* mRNA expression levels compared to phenotype of tumors.

**Phenotype**	**Significant variation in fold**	
***KIF14***	***E2F3***
Age at diagnosis (median 24 months)	0.01*	0.67
Duration (median 1.5 months)	0.34	0.55
Laterality	0.04*	0.28
Differentiation	0.31	0.91
Familial	0.63	0.19
Invasion	0.81	0.51

*There was a statistical increase in *KIF14* expression with older age at diagnosis (p=0.01) and in unilateral RB patients (p=0.04).

### Immunoreactivity of KIF14 in non-neoplastic retina and RB tumor cells

**Figure 2:**
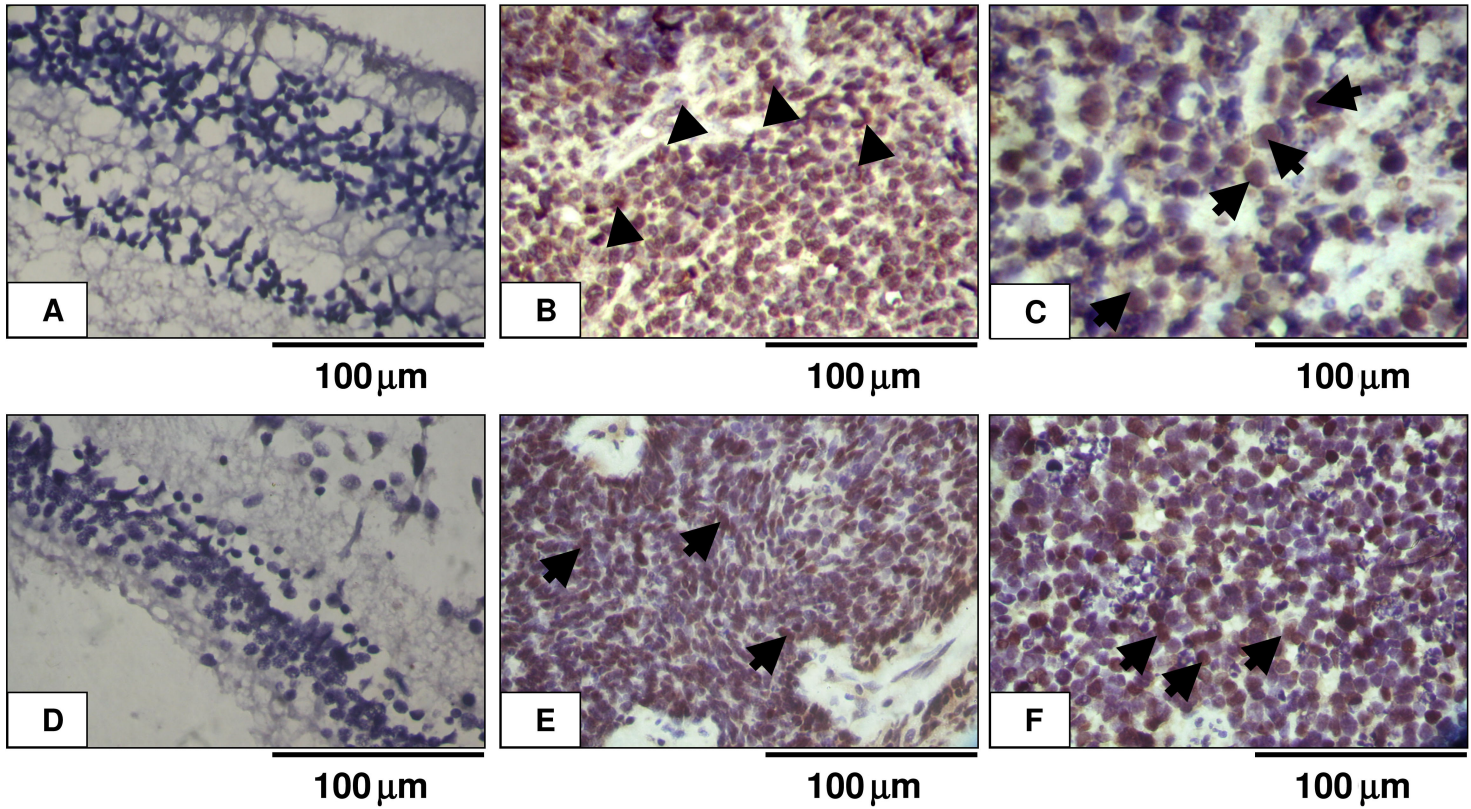
*KIF14* and *E2F3* expression in tumor and control retina. **A** and **D** show no immunoreactivity of *KIF14* and *E2F3* in non-neoplastic retina. **B** and **C** show positive nucleocytoplasm (arrow heads) and nuclear (arrows) expression of *KIF14* in tumor cells. **E** and **F** show positive nuclear (arrows) expression of *E2F3* in tumor cells. All are 3,3′-diaminobenzidine staining with hematoxylin counterstain. The pictures were taken under 40X magnification.

No immunoreactivity for KIF14 or E2F3 was noted in the control healthy retina (Figure 2A,D), whereas KIF14 expression was localized to the nucleus and cytoplasm in tumor cells (Figure 2B,C), and E2F3 expression was localized to the nucleus (Figure 2E,F). All 6 tumors, which were stained, showed heterogeneous expression of KIF14 (2+ staining) and E2F3 (3+ staining).

## Discussion

After knocking out the tumor suppressor *RB1* gene in the tumor-prone retinal cells, RB has to progress into full-fledged malignancy. This is achieved by triggering oncogenes, which can help in multiplication of the tumor cells. Different cancers take different routes for progression based on genetic instability. Comparative genomic hybridization and microarray-based PCRs have been narrowed to a few oncogenes, which probably helps RB to progress [11-14]. *KIF14* and *E2F3* may represent important pro-oncogenes, which have been seen to be deregulated in most RB tumors. By real time PCR, we found an overexpression of these 2 oncogenes in all tumors studied compared to fetal, age-matched and adult retinas. The sensitivity of real time PCR is perhaps higher compared to the previously used techniques to identify the amount and percentage of tumors, which showed deregulation in the genes studied. Nucleocytoplasmic localization of *KIF14* possibly represent its cytoplasm to the nucleus mobilization during spindle formation as shown by previous study [15]. This in addition to the nuclear localization of E2F3 may represent the functional intactness of both proteins in tumor cells. These 2 oncogenes act at different points in the cell cycle to accomplish proliferation after deregulation of pRB. E2F3 acts as a transcriptional activator of genes that are derepressed following transition through the RB1-dependent G_1_/S-phase cell cycle checkpoint [16], and play a role in DNA replication. KIF14 plays an important role during mitosis [6,15], and overexpression results in error-prone mitosis and tumor progression.

As seen with our earlier report [5], phenotype correlations revealed a statistically significant increase in mRNA expression of *KIF14* in patients at older age at diagnosis. There was a statistically significant increase in *KIF14* mRNA expression with unilateral RB compared to bilateral RB (p=0.04), which was not reported in our earlier study. As the median age at diagnosis of unilateral RB is late compared to bilateral RB, this correlates with a significant increase in the levels of *KIF14* mRNA expression in older age at presentation. Probably unilateral late onset tumors need more genetic instability for tumor progression, as retinal cells are in the final stage of terminal differentiation. *E2F3* mRNA expression levels did not show statistical significance with different phenotype variables. As *KIF14* and *E2F3* genes have a role in cell proliferation, and the severity of RB is related to the rate of proliferation of the tumor, even with known bias in the patient’s history, duration of the disease was calculated for individual patients and correlated with the relative *KIF14* and *E2F3 mRNA* expressions. No significant variability in *KIF14* and *E2F3* mRNA expression was noted with increase or decrease in disease duration.

The highly statistically significant decrease in the levels of *KIF14* mRNA expression (p<0.01) and *E2F3* mRNA expression (p<0.001) in chemotherapy-treated cases shows the effect of chemotherapy on proliferative tumor cells. This needs further validation in more numbers of chemotherapy treated tumor samples. In addition to the aforedescribed finding, the statistical increase in the mRNA expression of *E2F3* in fresh tumors compared to RB cell lines (p=0.01) discourages the use of RB cell lines to evaluate this gene for further studies.

This is the first report on the levels of two important candidate genes (*KIF14* and *E2F3*) for proliferation of RB tumor simultaneously done in a larger cohort with phenotype association.
